# IPEx: A gamification tool for learner application of pharmacologic principles of opioid use, misuse, and addiction

**DOI:** 10.1002/prp2.1141

**Published:** 2023-09-27

**Authors:** Kelly Karpa, Josie Ward, Melanie Stegman, Arthur Berg, Shou Ling Leong

**Affiliations:** ^1^ Department of Medical Education East Tennessee State University Quillen College of Medicine Johnson City Tennessee USA; ^2^ East Tennessee State University Quillen College of Medicine Johnson City Tennessee USA; ^3^ Maryland Institute College of Art Baltimore Maryland USA; ^4^ Department of Public Health Sciences Pennsylvania State University College of Medicine Hershey Pennsylvania USA; ^5^ Department of Family and Community Medicine Pennsylvania State University College of Medicine Hershey Pennsylvania USA

**Keywords:** gamification, opioid, pharmacology, pharmacy education, undergraduate medical education

## Abstract

Opioids are often prescribed to treat chronic pain ailments, despite lack of evidence for many conditions. Prescriptions frequently become the gateway to opioid misuse and abuse. In response to the opioid crisis, medical school educators in the state of Pennsylvania developed core competencies pertaining to opioids and addiction for which all medical students should demonstrate proficiency before graduation. To enable students to achieve these competencies, we developed a web‐based app (IPEx) that delivers a gamified experience for learners in which they are (re)exposed to opioid competencies and practice applying pharmacologic principles in the context of a series of longitudinal patient scenarios. Learning and application are measured by student responses to application questions before and after each of five modules. Prior to launching the IPEx tool broadly, we wished to test the application questions; thus, we invited fourth year medical students to complete a 45 question quiz based on IPEx module content. Students had no specific preparation prior to taking the quiz but had been exposed to all content elsewhere in the curriculum. A total of 45 of 141 medical students (32%) opted to complete the quiz (mean score was 47% ± 13%; range 18%–73%). Cronbach alpha for the instrument was .74. These results suggest that the instrument has internal validity, and medical students have room for growth when it comes to application of opioid related competencies, a situation that the IPEx tool may be uniquely suited to remedy.

## Graphical abstract

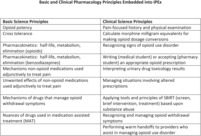

**Abbreviations**
IPEx: Interprofessional Experience App
MAT: medication assisted treatment
PDMP: Prescription Drug Monitoring Program
PPG: Pennsylvania Physician General

## 1 INTRODUCTION

Despite lack of evidence or proven efficacy, opioids are often prescribed to treat chronic pain.^1^ Moreover, abuse of opioids continues to surge.^2^ Overdose deaths involving opioids increased by 56% from 2019 to 2020.^3^ Combatting this situation will require a concerted effort of healthcare professionals as well as educators who are training the next generation of providers. In 2014, the Pennsylvania Physician General (PPG) created the Safe and Effective Prescribing Practices Task Force, which subsequently launched a second task force charged with developing core medical education competencies about opioids and addiction.^4^ In response to the ongoing public health crisis, these competencies were recently revised, with contributions from each of Pennsylvania's 10 medical schools.^5^

Physicians and pharmacists play pivotal roles in battling this crisis. However, students enrolled in medical school and pharmacy programs rarely have opportunities to learn and work together. Given the magnitude and complexity of opioid addiction, engaging these two professions in collaboration and dialogue could be beneficial as we develop solutions for this challenging situation. Students can learn with, from, and about the issues that each profession faces in the context of the opioid epidemic, enhancing interprofessional collaboration, and teamwork. Indeed, an interprofessional classroom‐based case discussion for medical and pharmacy learners that included a topic of opioid misuse received positive student feedback.^6^ While pre‐clinical case discussions are useful starting points for interprofessional student learning, they do not provide opportunities to teach/assess application skills or longitudinal management of patients with complex issues in clinically‐relevant contexts. To accomplish these objectives in a low stakes learning environment, interprofessional experience (IPEx) was created.

IPEx is a gamified learning tool that allows medical and pharmacy students to collaborate while applying pharmacologic principles in the context of a series of longitudinal patient scenarios. IPEx addresses each of the competencies outlined by the PPG taskforce as well as pharmacy competencies of patient care, pharmacotherapeutics, systems‐based care, communication, and professionalism *in the context of* interprofessional communication *and* patient management.^7^ Gamification is defined as the use of game design elements in non‐game contexts—such as education.^8^ IPEx incorporates 17 of 19 gaming attributes identified by Bedwell et al. that are believed to influence learning^9^ (Table 1). In healthcare, gamification shows promise for improving learning outcomes^10^, ^11^; yet, the true influence of gamification in medical education for learning, retention, and patient treatment must be investigated further.^12^

**TABLE 1:** Examples of gaming attributes embedded within IPEx.

Game attribute	IPEx characteristic
Adaptation	Students are permitted to make mistakes, but IPEx will correct them and explain the reasoning
Assessment	Correct/incorrect responses are tracked; student teams can, theoretically, “compete” with each other for the highest scores
Challenge	Students learn new information that they are challenged to apply to the scenario (e.g., converting opioid dosages, choosing the most appropriate therapy, managing complex issues, etc.). Correct/incorrect responses are tracked
Conflict	There are multiple conflict points between the patient and her healthcare providers that students must navigate. Best and sub‐optimal responses are featured and explained
Control	The learner is in control of making choices at multiple “decision points” throughout the experience
Interaction (interpersonal)	Interactions between pharmacy and medical students are not only encouraged—they are mandatory at some checkpoints in the game
Interaction (social)	Pairing of a pharmacy and medical student learner as a team encourages a sense of belonging
Communication	Communications are reflective of “electronic messaging” in electronic health records
Location	The video settings evoke realism in the context of visits to pharmacy and medical offices
Mystery	There is a gap between existing knowledge of students when they begin and the discrepancies, surprises, and complexity of the situations they encounter
Players	Students are assigned an avatar name when they log into the game
Progress	Student's progress throughout is apparent by the context of the module headings and subheadings in the Table of Contents
Surprise	There are random elements of surprise throughout the experience
Representation	The player's reality is represented by the context of the profession in which they are training (e.g., medical students have a different iteration of the experience compared to pharmacy students)
Rules/goals	Well defined guidelines are provided at the beginning of the experience (e.g., conduct a *focused* history and physical exam; method of communicating via e‐messaging, etc.)
Safety	The experience provides a safe place to make mistakes and learn from them
Sensory stimuli	There are a variety of active and interactive components to maintain auditory and visual engagement (e.g., multiple choice questions, videos, readings, decision points, partner interactions, etc.)

Abbreviation: IPEx, Interprofessional Experience App.

One key attribute of gamification is realism, where a game attempts to accurately mimic real life through gameplay mechanics and visuals; IPEx strives to be as true to life as possible. Within the five IPEx modules are dozens of required readings with “factual” information that students must apply throughout the experience. Videos depict patients and family members in medical and pharmacy settings; e‐messaging between medical and pharmacy students mimics provider communication within an electronic medical record; medical charts are updated in real‐time based upon decisions students make; and a Prescription Drug Monitoring Program (PDMP) database as well as a Pharmacy Profile update instantaneously as care decisions are made. Students are tasked with timely decision making and interprofessional accountability; they receive immediate feedback on both correct and incorrect responses/decisions; sometimes, they are permitted to “try again” before being corrected (e.g., when writing an opioid prescription). Additionally, student knowledge before, during, and after each module is assessed through multiple choice and short answer questions to determine the effectiveness of the tool in meeting learning objectives. The IPEx opioid learning tool incorporates the discipline of pharmacology as both a basic and clinical science (Table 2). A control iteration of IPEx that only uses pre‐ and post‐assessments and readings, but lacks all interactive and patient‐specific components was also designed, allowing for comparisons of learning/application resulting from active engagement with the tool and a partner.

**TABLE 2:** Basic and clinical pharmacology principles embedded into IPEx.

Basic science principles	Clinical science principles
Opioid potency	Pain‐focused history and physical examination
Cross tolerance	Calculate morphine milligram equivalents for making opioid dosage conversions
Pharmacokinetics: half‐life, metabolism, elimination (opioids)	Recognizing signs of opioid use disorder
Pharmacokinetics: half‐life, metabolism, elimination (benzodiazepines)	Writing (medical student) or accepting (pharmacy student) an appropriate opioid prescription
Mechanisms non‐opioid medications used adjunctively to treat pain	Interpreting urinary drug toxicology results
Unwanted effects of non‐opioid medications used adjunctively to treat pain	Managing situations involving altered prescriptions
Mechanisms of drugs that manage opioid withdrawal symptoms	Applying tools and principles of SBIRT (screen, brief intervention, referral, treatment) based upon substance abuse
Nuances of drugs used in medication assisted treatment (MAT)	Recognizing and managing opioid withdrawal symptoms
	Performing warm handoffs to providers who assist in managing opioid use disorder

Abbreviation: IPEx, Interprofessional Experience App.

Given that the primary goal of IPEx is to help students (especially future prescribers) *apply principles* related to pain/opioid management, we needed to be certain that the application questions used to gauge efficacy of the tool were written at an appropriate level. Specifically, if students scored high on the pre‐module assessments (e.g., indicating that they already *know* and can *apply* all the information), there is little/no room for growth to be captured on the post‐module assessments. Thus, our objective was to ascertain fourth year medical student performance on the IPEx assessment questions, without any explicit prior preparation. We hypothesized that there would be room for improvement.

## 2 METHODS

### 2.1 Development of the IPEx tool

Case content, including assessments, was developed by a pharmacist and pharmacologist, in conjunction with a physician practicing at a Family Medicine site. Development of the IPEx app was coded and designed in Unity3D and deployed as a WebGL on an Azure server (Microsoft). The app receives its information from easily‐modifiable Google spreadsheets that are uploaded and converted to tab‐separated values.

### 2.2 Item assessment

To assess the knowledge and application skills of fourth year medical students, learners at our medical school were invited to complete a 45‐question quiz. The invitation took place in‐person, during a required clerkship activity at the transition between their third and fourth years of medical school; thus, students had completed all required pre‐clinical and all required clerkships.

Interested students completed the quiz in REDCap. Participation in the quiz was voluntary, and student responses to quiz questions were anonymous. Student responses were subsequently converted to “correct” or “incorrect” answers.

### 2.3 Statistics

Individual item analysis was conducted using the “tab_itemscale” function in the R package sjPlot to calculate item discrimination and item difficulty. Mean scores ± standard deviation were calculated for each student. Cronbach's alpha measured the internal consistency of the test. To test correlations between seven pairs of questions assessing the same concept in two different ways, we used the Pearson's product–moment correlation test implemented in the “cor.test” function in R.

## 3 RESULTS

All 45 quiz questions were voluntarily completed by 45 of 141 MD students (32%). Mean quiz score for learners was 47% (SD 13%; range 18%–73%). Only one student scored >70%. Cronbach alpha for the overall instrument was .74, indicating acceptable internal consistency (>.7).

## 4 DISCUSSION

Results of student performance are in line with what we had anticipated. Despite students having already completed preclinical curricula and all required clerkships—where they had been exposed to basic and clinical pharmacology related to opioid use/misuse—there appears to be room for improvement with respect to applying that information and knowledge in the context of patient scenarios. Fourteen questions were specifically crafted to test for correlations (e.g., testing the same concept, but from different angles), but no correlations were seen in student responses to these questions; this suggests that even correctly‐answered questions were at least partial “guesses” as opposed to being answered correctly because of true knowledge application. Examples of items that had a high percentage of correct responses and a high discrimination index included (a) a synthesis question that required students to recognize the Drug Enforcement Administration (DEA) classification of a scheduled pain medication and know how many refills were appropriate, as well as (b) recognizing red flag behaviors that suggest a patient may be misusing/abusing opioids. On the other hand, items that had a low percent correct and poor discrimination index included items that (a) required identifying which drug had been utilized, based upon metabolites identified in a urine toxicology screen and (b) recognizing that kidney damage may occur with non‐steroidal anti‐inflammatory drugs.

While student performance was not stellar, it is important to remember that students had not prepared in any way for this assessment. It was administered “cold,” in effort to determine if the answers to the questions we crafted would already be “known” to all the learners. These results seem to support our hypothesis that there is room for improvement with regard to students' knowledge and/or application of competencies related to opioid use/misuse. This analysis confirms that students will not know all the information presented in IPEx *a priori* simply by completing required coursework. Thus, the IPEx tool could be adopted by medical schools to improve student application of opioid competencies. Having students complete the quiz before launching the IPEx tool was helpful to us as educators in other ways; when reviewing individual item statistics, we determined that one of the questions was unknowingly written with two potential correct responses. This oversight has been corrected.

Even though the tool was explicitly crafted with MD and PharmD students from the state of Pennsylvania in mind, we recently had the opportunity to beta test the tool with four pairs of partnered students, including pharmacy and medical student participants in the states of Pennsylvania, Tennessee, and Virginia. Students provided valuable insight about ways to improve the learner experience with the game (e.g., suggestions to improve the e‐messaging component) and unanimously indicated that the game is applicable to the clinical years of training (rather than preclinical). Beta testers commented about the learning experience with this app being ideal; enjoying the short explanations and content; the modules being short and straight to the point which made it easy to understand; and indicated that they learned quite a bit. Given the enthusiastic endorsement, we believe most of the concepts are relevant to learners regardless of their geographic location. While laws, statutes, and regulations concerning prescribing and dispensing of opioids differ by state, there are enough similarities regarding these concepts under federal laws that the tool has broad applicability for teaching clinical applications to students in a low‐stakes environment, while simultaneously promoting interprofessional learning.

Since IPEx was originally crafted with learners from the state of Pennsylvania in mind, this could be viewed as a limitation to the general applicability of the tool. However, we have tried to mitigate this by explicitly emphasizing within the tool the concepts that differ on a state‐by‐state basis. Nonetheless, there are certainly state‐specific laws of which we are unaware. At the present time, the IPEx tool is not configured to recognize nuances of nurse practitioners and physician assistant opioid prescribing regulations; these learners would need to follow rules for physician prescribers if using the tool. A potential limitation to the data reported herein may pertain to the motivation of students that opted to complete the quiz questions; answers were submitted anonymously and did not count toward a grade.

The user interface of IPEx is designed to be simple to navigate, allowing users to feel in control, while simultaneously presenting complex information and situations. Because the app is designed for students who are highly motivated and who have little time, the tool was designed without complex game mechanics, mimicking innate encounters with patients and other healthcare providers. The IPEx records student choices, e‐messages between partners, and chart notes that students write. This information is stored on an Azure server and can be provided to course instructors who wish to assign grades based upon student performance. Importantly, IPEx can be altered easily by instructors to modify written text, number of modules, sequence of events, videos, and images simply by changing the content of Google spreadsheets and the Azure server. This makes the IPEx platform ideal as a learning tool because it can be modified to allow student collaborations in any content area.

The next step in this project is recruiting medical and pharmacy students for randomization to the gamified or control iteration of the IPEx tool. Having cohorts of students assigned to the active/interactive iteration and the control version will improve understanding of how gamification elements impact student learning, while simultaneously improving knowledge and application of crucial principles related to chronic pain/opioid use/misuse and interprofessional interactions. Medical and pharmacy school faculty who would like to have their students participate in this learning experience can contact the authors for additional enrollment details.

## AUTHOR CONTRIBUTIONS

Kelly Karpa conceived and designed the project, Melanie Stegman developed the software, Josie Ward and Kelly Karpa wrote the original manuscript draft, Arthur Berg analyzed the data, Melanie Stegman and Shou Ling Leong edited drafts of the manuscript, and Shou Ling Leong acquired funding.

## CONFLICT OF INTEREST STATEMENT

The authors declare no conflicts of interest.

## ETHICS STATEMENT

This work was conducted under approval of the Institutional Review Board at our institution under protocol #13887.

## Data Availability

The data that support the findings of this study are available from the corresponding author upon reasonable request.
